# Sexual and Vertical Transmission of Zika Virus in anti-interferon receptor-treated Rag1-deficient mice

**DOI:** 10.1038/s41598-017-07099-7

**Published:** 2017-08-03

**Authors:** Clayton W. Winkler, Tyson A. Woods, Rebecca Rosenke, Dana P. Scott, Sonja M. Best, Karin E. Peterson

**Affiliations:** 1 Laboratory of Persistent Viral Diseases, Rocky Mountain Laboratories, National Institute of Allergy and Infectious Diseases (NIAID), National Institutes of Health (NIH), Hamilton, MT 59840 USA; 2 Rocky Mountain Veterinary Branch, Rocky Mountain Laboratories, National Institute of Allergy and Infectious Diseases (NIAID), National Institutes of Health (NIH), Hamilton, MT 59840 USA; 3 Laboratory of Virology, Rocky Mountain Laboratories, National Institute of Allergy and Infectious Diseases (NIAID), National Institutes of Health (NIH), Hamilton, MT 59840 USA

## Abstract

Although Zika virus (ZIKV) is primarily transmitted to humans by the *Aedes aegypti* mosquito, human-to-human transmission has also been observed from males-to-females as well as mother-to-offspring. In the current study, we studied both sexual transmission (STx) and vertical transmission (VTx) of ZIKV using anti-IFNAR1-treatment of *Rag1* ^−/−^ (AIR) mice. These mice have suppressed type I IFN responses and lack adaptive immune responses, leading to a prolonged infection prior to clinical disease. STx of ZIKV from infected AIR males to naive *Ifnar1* ^−/−^ females was observed with greater than 50% incidence, with infection observed in the vaginal tract at early time points. In the case of a resulting pregnancy, virus was also found in the uterus and placental tissue. In additional studies, VTx of virus was observed in AIR female mice. Specifically, peripheral ZIKV infection of pregnant AIR females resulted in detectable virus in brain and/or lymph nodes of fetuses and/or pups. VTx of ZIKV was stochastic, in that not all fetuses/pups within the same dam had detectable virus and infection was not associated with breakdown of maternal/fetal placental barrier. This provides a new model to study the barriers to STx and VTx of ZIKV and the immune responses essential to preventing transmission.

## Introduction

Zika Virus (ZIKV) is a flavivirus, originally isolated in Uganda, but more recently associated with outbreaks in both South and North America. ZIKV infection in humans is normally asymptomatic or has mild symptoms of rash, fever and conjunctivitis^1^. However, ZIKV infection has led to the development of Guillain-Barré Syndrome (GBS) in a small number of people and can result in perinatal issues including fetal insufficiency, fetal death, microcephaly and post-natal microcephaly in pregnant women^2–4^. Thus, the most severe diseases associated with ZIKV infection involve dysfunction of the nervous system, including impaired development following infection of the central nervous system (CNS) in the developing fetus.

ZIKV is primarily transmitted to humans by the *Aedes aegypti* mosquito. However, ZIKV is also transmitted human-to-human by sexual transmission (STx) from male- to- female and by vertical transmission (VTx) from mother-to-fetus. Infectious ZIKV has been reported in human seminal fluid and direct male-to-female STx of ZIKV in humans has been confirmed^5–7^. However, important questions remain about whether STx has any influence on VTx, what specific barriers inhibit STx and VTx of virus and how they function, as well as the specific roles of innate and adaptive immune responses in regulating STx and VTx. Thus, small animal models of viral transmission will be essential to understand mechanism and develop therapeutics.

Modeling ZIKV infection and transmission in small animal models such as mice has remained difficult due in part to the strong type I interferon (IFN) response to ZIKV in mice. In humans, ZIKV suppresses the type I IFN response by antagonism of the STAT2 adaptor protein, likely allowing for greater viral replication^8, 9^. Mice deficient in the type I IFN receptor (*Ifnar1* ^−/−^) develop high viral titers when infected with ZIKV and severe disease including a dramatic loss in body weight within 5–7 days post infection (dpi)^8^. IFN receptor knockout mice have been utilized to study ZIKV transmission. STx was recently shown between males and females in IFN α/β and -γ receptor knockout mice, which are both type I and II IFN signaling incompetent^10^. VTx of ZIKV has been observed in *Ifnar1* ^−/−^ mice, with infection inducing dramatic fetal insufficiency by 13–15 days of embryonic development^11^. These studies indicate that the IFN response is critical for preventing STx and VTx. However, further studies are needed to demonstrate the potential for STx and VTx in models where the IFN response is suppressed rather than incompetent, as is the case in humans. For example, treatment of wildtype (WT) C57BL/6 mice with anti-IFNAR antibody (MAR1-5A3) also results in VTx, albeit at lower levels of virus infection in the fetus^11^. Thus suppression, rather than complete abrogation, of the type I IFN response may more accurately reflect ZIKV infection and transmission in humans. Additionally, suppression, but not abrogation, of the IFN response allows the analysis of how other components of the immune response may influence virus transmission.

We recently described a mouse model of ZIKV infection using αIFNAR1 antibody treated *Rag*1^−/−^ (AIR) mice^12^. Although *Rag1* ^−/−^ mice alone are not susceptible to ZIKV infection, AIR mice are susceptible, with virus infection of brain and testes. Disease progression is slower in AIR mice compared to other mouse models permissive to ZIKV infection^8, 13, 14^, with initial weight loss observed around 9–12 dpi and severe weight loss not observed until 14–17 dpi ^12^. In comparison, ZIKV-infected *Ifnar1* ^−/−^ mice have severe weight loss by 6–7 dpi^8^. This delay in disease onset in AIR mice provides a longer window to study transmission of virus particularly during the earlier stages of virus infection. We therefore examined AIR mice for VTx and STx of ZIKV. Pregnant female AIR mice could transmit virus vertically to the fetus, with approximately 26% of fetuses/pups having detectable virus in the brain. Additionally, we demonstrated STx of virus from ZIKV-infected AIR male to *Ifnar1* ^−/−^ female mice. Transmission primarily occurred during a specific time frame post-infection in males, suggesting efficiency of STx is finite.

## Results

### STx of ZIKV from AIR mice to *Ifnar1*^−/−^ mice

**Figure 1:**
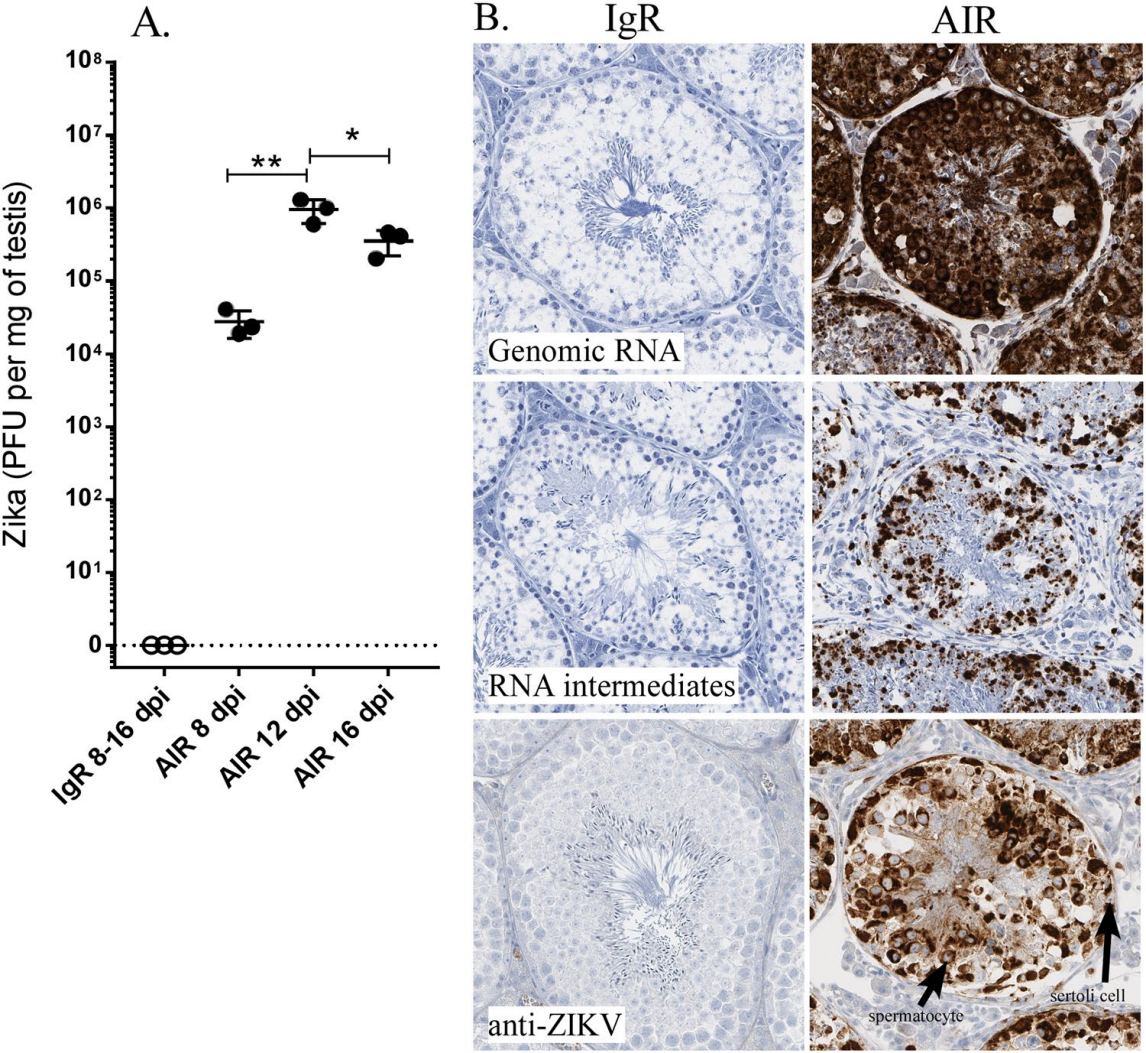
ZIKV infection in the testes of AIR mice. (**A**) Plaque-forming units (PFU) of ZIKV in testes of infected normal mouse IgG-treated *Rag1* ^−/−^ (IgR) and AIR treated mice at 8, 12 and 16 dpi, detected as described in the methods. No virus was detected in the testes of IgR-treated mice. Statistical analysis was completed by One-way ANOVA, with Tukey post-test. *P < 0.05, **P < 0.01. (**B**) ISH labeling for (top panels) genomic RNA, (middle panels) replicative RNA intermediates and immunohistochemical labeling for ZIKV (bottom panels) in the testes of infected IgR and AIR mice.

In a previous study, analysis of ZIKV infected AIR mice at the clinical time point, 14–17 dpi, showed virus infection of the brain and testes by *in situ* hybridization (ISH) and immunohistochemistry (IHC) staining^12^. To determine if virus was present in the testes of AIR mice prior to, or at the onset of clinical disease, testes were analyzed for infectious virus by plaque assay. Virus titers peaked at 12 dpi with a slight decrease by approximately ½ a log by 16 dpi (Fig. 1A). ISH and IHC in the testes of infected AIR mice demonstrated infection and replication primarily in germinal spermatogonia and primary spermatocytes and to a lesser extent sertoli stromal cells, which was not observed in IgR mice (Fig. 1B). Thus, infected AIR male mice have infectious virus in the testes early during infection.

**Figure 2:**
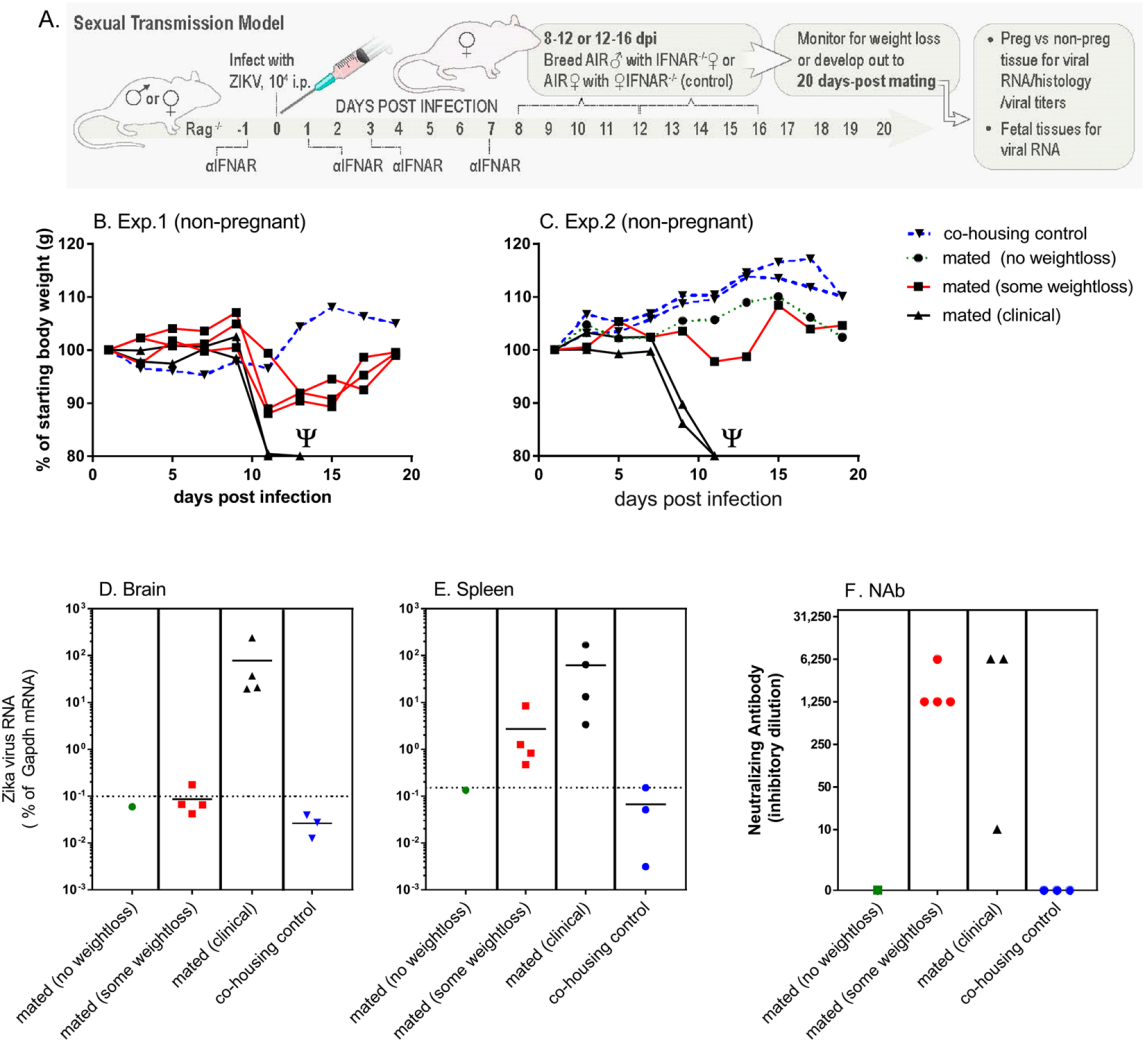
STx of ZIKV in AIR mice. (**A**) In two replicate experiments, AIR male and female mice were infected with ZIKV intraperitoneally (i.p.). At 8 dpi, these male and female AIR mice were mated/co-housed with *Ifnar1* ^−/−^ female mice for 4 days at the times indicated. (**B**,**C**) *Ifnar1* ^−/−^ female mice were then monitored for pregnancy and weight loss for 20 days post mating (dpm). Data are shown as individual percent starting body weight of *Ifnar1* ^−/−^ females bred with AIR mice. Plots are color-coded to indicate weight loss groups. Ψ indicates mice euthanized due to clinical disease. Two mice became pregnant during these experiments and are shown in Supplementary Figure 3. (**D–F**) At 20 dpi, (**D**) brain, (**E**) spleen and (**F**) plasma from *Ifnar1* ^−/−^ mice were analyzed for viral RNA or NAb. (**D–E**) quantitative real-time (qRT) PCR analysis of individual mice for viral RNA in (**D**) brain and (**E**) spleen. Dotted line indicates sensitivity level of assay for each tissue. (**F**) Inhibitory dilution of neutralizing antibody (NAb) for each animal. No NAb titer was detected for the mouse that was mated with no weight loss (green symbol) or co-housing control mice. One mouse could not be tested in the mated (clinical) group due to lack of plasma sample.

To determine if ZIKV could be transmitted sexually, ZIKV-infected male AIR mice were mated with naïve *Ifnar1* ^−/−^ females between 8–12 dpi or 12–16 dpi (Fig. 2A). As a control for incidental transmission, *Ifnar1* ^−/−^ female mice were co-housed with ZIKV-infected AIR female mice. Following mating, males were removed and *Ifnar1* ^−/−^ females were weighed every other day as a measure of clinical disease. Of the 11 *Ifnar1* ^−/−^ females mated with the AIR mice at 8–12 dpi, two became pregnant and gained weight throughout the experiment (Supplementary Figure 1A). Four mice (36.4%) lost more than 20% of starting body weight indicating clinical disease (Fig. 2B,C: black lines, Ψ indicates euthanized). Another four mice (36.4%) had initial weight loss beginning between 7–9 dpi, but regained weight by the experimental endpoint (red lines). These mice never lost more than 10% of starting body weight. One mouse did not lose weight (dotted green line). Thus, 8 of 11 (72%) *Ifnar1* ^−/−^ female mice bred with ZIKV-infected AIR male mice that did not become pregnant showed some form of weight loss, suggesting sexual transmission of ZIKV. In contrast, none of the *Ifnar1* ^−/−^ mice co-housed with female AIR mice lost weight (Fig. 2B,C: dashed blue lines).

Additional *Ifnar1* ^−/−^ females were mated with the same AIR males in Exp. 1, but a later point (12–16 dpi) to determine if virus could also be transmitted at later stages of infection. Interestingly, none of these *Ifnar1* ^−/−^ females lost weight (Supplementary Fig. 1C). Furthermore, they did not have detectable virus in any tissue examined (data not shown). One mouse failed to gain weight throughout the experiment (Supplementary Fig. 1C, green symbols) and one mouse did become pregnant and had normal weight gain (Supplementary Fig. 1C, red symbols). Thus, ZIKV-infected AIR male mice can sexually transmit virus to *Ifnar1* ^−/−^ female mice, but transmission occurs primarily during the early 8–12 dpi time point.

### Detection of ZIKV RNA and NAb in non-clinical *Ifnar1*^−/−^ females following STx

To confirm ZIKV infection in female *Ifnar1* ^−/−^ mice, brain and spleen tissue was assayed for ZIKV RNA by qRT PCR and plasma viremia and neutralizing antibody (NAb) was measured (Fig. 2D–F). Clinical *Ifnar1* ^−/−^ mice that lost greater than 20% starting body weight (black symbols) had high levels of viral RNA in brain and spleen tissue, correlating with ZIKV infection (Fig. 2D,E). Interestingly, *Ifnar1* ^−/−^ mice that showed initial weight loss (red symbols) had detectable viral RNA in spleens, albeit 10–100 fold lower than clinical mice (Fig. 2E). Virus was not detected in spleens from the no weight loss *Ifnar1* ^−/−^ mouse or from control mice (data not shown). In correlation with detectable virus, *Ifnar1* ^−/−^ mice with clinical disease or initial weight loss had high levels of NAbs against ZIKV (Fig. 2F). In contrast, the mouse with no weight loss (Fig. 2C, dotted green line) and co-housing control mice (Fig. 2B,C dashed blue line) had no ZIKV NAb (Fig. 2F), suggesting they were never infected. Additionally, one of the two pregnant mice from these experiments (Supplementary Fig. 1B) had a detectable NAb response. None of the mice analyzed had viremia at the time of sampling (data not shown). Together, these findings suggest that even in mice that did not develop severe clinical disease, ZIKV infection was detectable as indicated by initial weight loss and viral RNA in the spleen. The presence of NAb was also suggestive of infection, although not conclusive as exposure to viral antigen may also initiate a response.

*Ifnar1* ^−/−^ mice bred to infected AIR males at 12–16 dpi did not lose weight (Supplementary Fig. 1C), however, the mouse that failed to gain weight (Supplementary Fig. 1C,D green symbols) and the mouse that became pregnant (Supplementary Fig. 1C,D red symbols) had detectable NAb indicating they were infected at a low titer or were exposed to ZIKV antigen. Collectively, these experiments suggest that STx occurs with high frequency in this model at the early time point (9 of 13 mice at 8–12 dpi, including pregnant mice), and less frequently at the later time point (2 of 6 mice at 12–16 dpi). Interestingly, some of the *Ifnar1* ^−/−^ mice infected with virus by STx controlled virus replication after initial weight loss, indicating that this route of infection can be suppressed in the absence of type I IFNs.

### ZIKV infection of vaginal tract correlates with development of clinical disease

**Figure 3:**
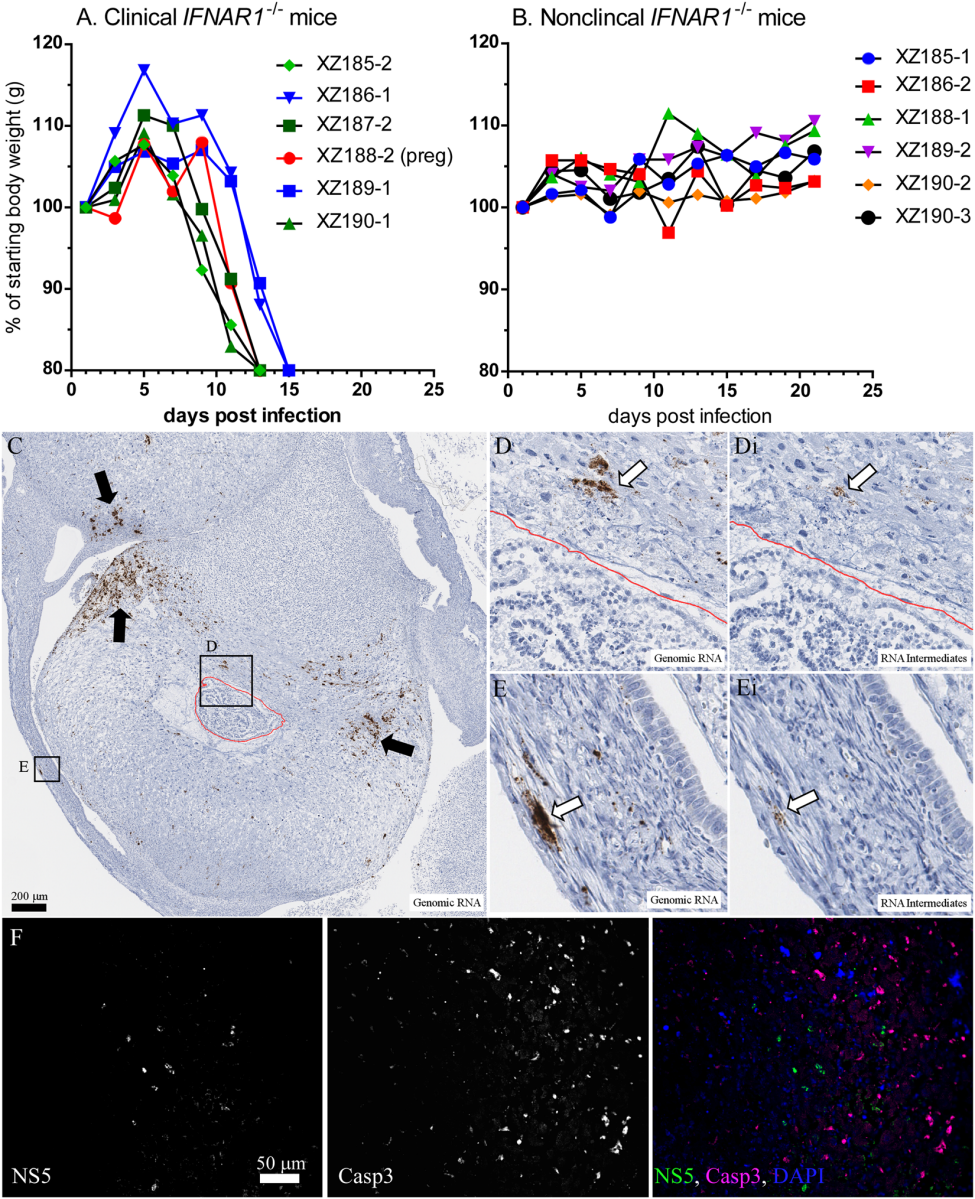
Detection of virus in vaginal tissues following STx. (**A–B**) Weights of (**A**) clinical and (**B**) non-clinical mice described in Table 1. Data are shown as individual percent starting body weight, similar to that shown in Fig. 2B–C. (**A**) Mouse in red symbol/line (XZ188-2) was determined to be pregnant at the time of clinical disease. (**C–E**) ISH and (**F**) IHC of (**C,E**) uterus and (**C,D,F**) placental tissue from XZ188-2. (**C**) Low magnification of uterus, placenta and fetus from XZ188-2 stained for genomic RNA (brown). Red line is drawn to indicate fetal tissue, which was not positive for either genomic or intermediate viral RNA. (**D–E**) Higher magnification of (**D,Di**) placental and fetal or (**E,Ei**) uterus tissue showing detection of ZIKV (**D,E**) genomic RNA or (**Di,Ei**) intermediate RNA. Intermediate sections were stained in adjacent sections to those used for genomic RNA. (**F**) Placenta tissue from XZ188-2 showing viral protein (NS5) as well as active Caspase 3 staining (Casp 3) in localized areas of the placenta.

We next analyzed whether ZIKV could be detected in the vaginal tract following STx. 12 naïve female *Ifnar1* ^−/−^ mice were mated with ZIKV-infected AIR mice at 8–12 dpi. Vaginal swabs taken at 0, 5, 9–10 and 12–14 days post mating (dpm) were analyzed by real-time PCR to determine if virus could be detected in the vaginal tract. At 5 dpm, four mice were positive for virus (Table 1), with two swabs showing detectable viral RNA (+), and two swabs having viral RNA equivalent to 10^3^–10^4^ PFUs (++). By 9–10 dpi, 3 of these 4 mice remained positive for virus in the vaginal swabs and showed signs of clinical disease (Fig. 3A, black, red lines). An additional two mice were positive for virus at 9–10 dpm, but did not shown clinical signs until 12 dpm (Fig. 3A, blue lines). One mouse that did not develop clinical disease, but had a detectable NAb response (Table 1, XZ186-2) experienced weight loss at 11 dpm but recovered (Fig. 3B, red squares). All other mated *Ifnar1* ^−/−^ female mice did not demonstrate remarkable weight loss (Fig. 3B). Thus, positive vaginal swabs were a strong indicator for development of clinical signs with 6 of 7 mice that had positive swabs developing clinical disease (Table 1). These data also show that STx of ZIKV is associated with detectable virus in the vaginal tract, which strongly correlated with the development of clinical disease.

**Table 1:** Detection of virus in vaginal swabs of *Ifnar1* ^−/−^ mice mated to ZIKV-infected AIR males.

Mouse #^a^	0^b^	5	9–10 ^c^	12–14 ^d^	Clinical^e^	Viremia^f^	NAb^g^
XZ185-1	−	−	−	−	−	0	0
XZ185-2	−	+	−	na	9	0	1:6,250
XZ186-1	−	−	++	++	12	0	1:6,250
XZ186-2	−	−	++	−	−	0	1:1,250
XZ187-2	−	++	+	na	9	0	1:6,250
XZ188-1	−	−	−	−	−	0	0
*XZ188-2*	−	*+*	*+*	*na*	*10*	*0*	*1:6,250*
XZ189-1	−	−	+	−	12	0	1:6,250
XZ189-2	−	−	−	−	−	0	0
XZ190-1	−	++	++	na	10	0	1:6,250
XZ190-2	−	−	−	−	−	0	0
XZ190-3	−	−	−	−	−	0	0

^a^Italicized mouse (*XZ188-2*) was impregnated by an infected AIR male (Fig. 3).

^b^Vaginal swabs from mice at indicated dpm were analyzed for virus by RNA as described in methods. – indicates that CT values for viral RNA were comparable to mock controls (no detection below 29 cycles).; +indicates low detection of virus with CT values between 26–29; ++ indicates significant viral RNA with CT values less than 26.

^c^All mice were analyzed at 10 dpm except XZ185-2 and XZ187-2 which were analyzed at 9 dpm.

^d^Mice were analyzed at 14 dpm except XZ186-1, which was analyzed at 12 dpm, and the four mice that had already developed clinical disease and were no longer in the study (NA).

^e^dpi when clinical disease was observed; (−) not clinical.

^f^Viremia was tested for in all mice, but was not detected.

^g^NAb titers in plasma from indicated mice. Serial 5 fold-dilutions were completed. Highest dilution that inhibited 50% of virus infection is reported.

### Pregnancy associated with STx of ZIKV can lead to infection of uterine and placenta tissue

Of the 30 *Ifnar1* ^−/−^ females mated to ZIKV-infected AIR males, four mice became pregnant (Supplementary Fig. 1A,C). Three of the four pregnant mice had NAb against ZIKV (Supplementary Fig. 1B,D), but only one mouse, (XZ188-2, Exp. 3) showed signs of clinical disease. Analysis of the uterus from this mouse at the time of clinical disease showed detectable genomic and intermediate viral RNA in cells in the uterus (Fig. 3C,E,Ei), and placenta (Fig. 3C,D,Di,). IHC analysis also demonstrated numerous apoptotic cells (Fig. 3F) in areas of virus infection in the placenta. Fetal tissue was also found (Fig. 3C,D, red line), but there was no clear evidence of infection (Fig. 3C,D,Di). Two of the other pregnant mice had detectable NAb to ZIKV, despite a lack of clinical disease, indicating that they were at least exposed to virus antigen (Supplementary Fig. 1B–D). However, further analysis of these mice indicated no viremia and that viral RNA was not detected in the brain or spleen of the animals or in the placenta or brain of the resulting pups (data not shown). Thus, in the relatively few cases of pregnancy from ZIKV-infected AIR male mice, there was no clear demonstration of VTx. However, there was evidence of infection of both the uterus and the placenta. Therefore, ZIKV infection in AIR male mice leads to sufficient levels of virus in the testes that results in STx of virus and can result in infection of maternal tissues, including reproductive tissues.

### VTx of ZIKV to fetuses in AIR female mice

**Figure 4:**
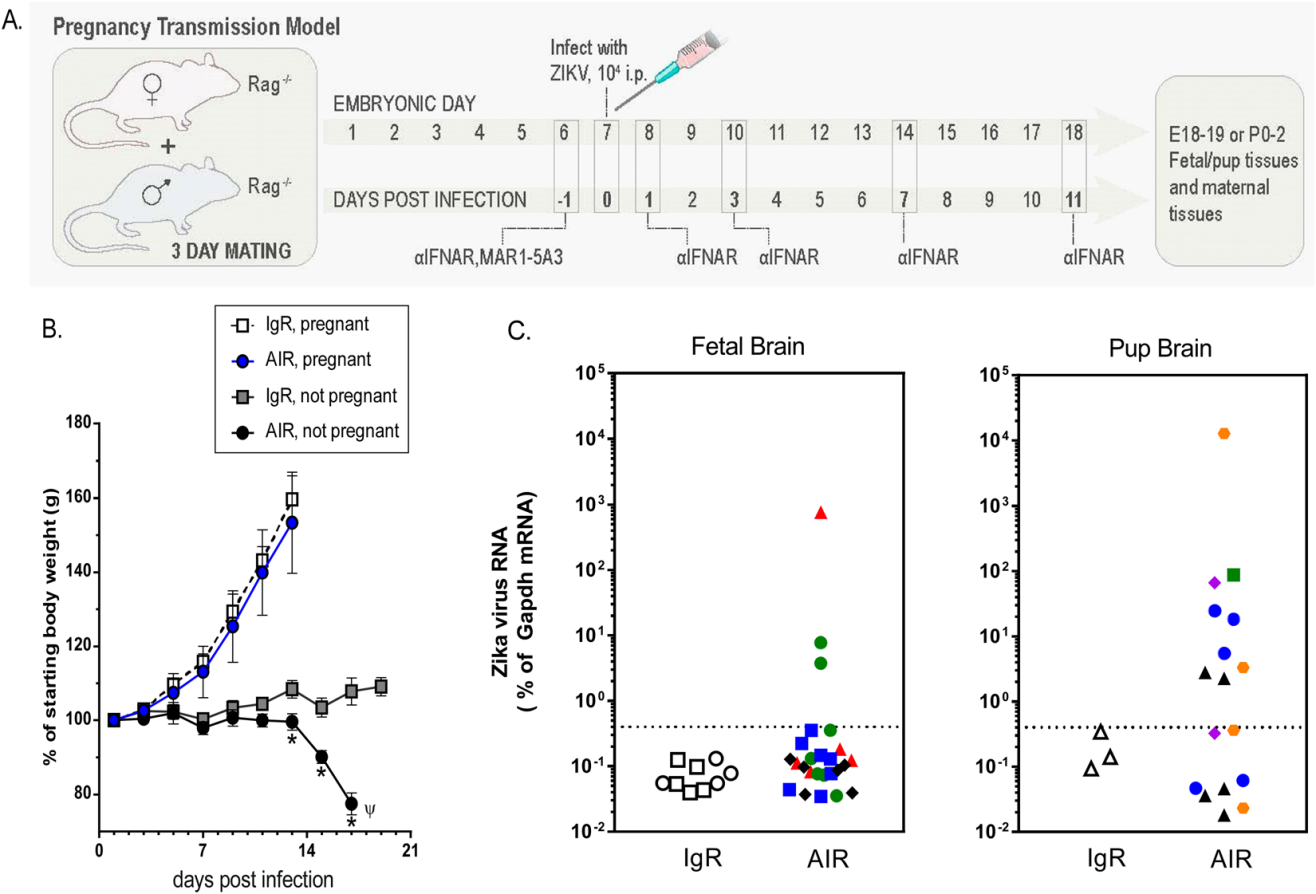
VTx of ZIKV in AIR mice. (**A**) *Rag1* ^−/−^ mice were bred for 3 days and infected with 10^4^ ZIKV i.p. at 7 dpm. Mice were treated i.p. with 1 mg of either normal mouse IgG or αIFNAR1 antibody on −1, 1, 3, 7 and 11 dpi and followed for weight loss and for delivery of pups. (**B**) Percent of starting body weight of ZIKV infected IgR or AIR non-pregnant or pregnant mice. (**C**) qRT PCR analysis of brain tissue from (left graph) fetal or (right graph) neonatal mice from pregnant mice in (**B**). The different symbol shapes/colors indicate individual litters.

In addition to the potential of VTx transmission of virus to the fetus following STx, VTx of ZIKV can also occur following peripheral infection of ZIKV. To examine what effect IFN suppression and deficiency of the adaptive immune response had on VTx following peripheral infection, we infected female AIR mice impregnated by *Rag1* ^−/−^ males with ZIKV at 7 dpm (Fig. 4A). Pregnant, ZIKV-infected AIR mice had similar weight gains as pregnant, ZIKV-infected IgR mice (Fig. 4B). Non-pregnant AIR mice started showing initial weight loss at ~12–13 dpi, with a 20% loss in weight (clinical disease) by 17 dpi (Fig. 4B). Thus, ZIKV-infection did not appear to dramatically affect pregnancy-induced weight gain in AIR mice, allowing for analysis of how ZIKV affects fetal development without complications of severe weight loss in the dam.

To determine if VTx occurred in this model, three litters from IgR mice and eight litters from AIR pregnant mice (Fig. 1B) were analyzed for VTx of ZIKV. Two litters from IgR and four litters from AIR mice were analyzed prior to birth at 11–13 dpi (approximately embryonic day18-19) and one and four litters, respectively, were analyzed post birth (14–17 dpi, post-birth days 1–3). Visually, all fetuses/pups were unremarkable in terms of overall size and head size. Three pups per litter were used for histological analysis and the remaining pups used for quantitative real-time (qRT) PCR detection of viral RNA. Analysis of brain tissue showed 3 of 26 (12%) of the fetuses (Fig. 4C) and 9 of 17 (53%) of the born pups (Fig. 4C), were positive for ZIKV RNA. Interestingly, not all fetuses/pups from the same litter were positive for virus (Fig. 4C, pups in same litter designated with same symbol). In total, 11 of 43 (26%) brains from fetuses/pups were positive for ZIKV RNA (Fig. 4C). These results indicate that ZIKV can be transmitted from dam to fetus in this model, but that the occurrence of VTx varies between fetuses in the same dam.

### Virus detection in CNS and lymph nodes of fetuses following VTx

**Figure 5:**
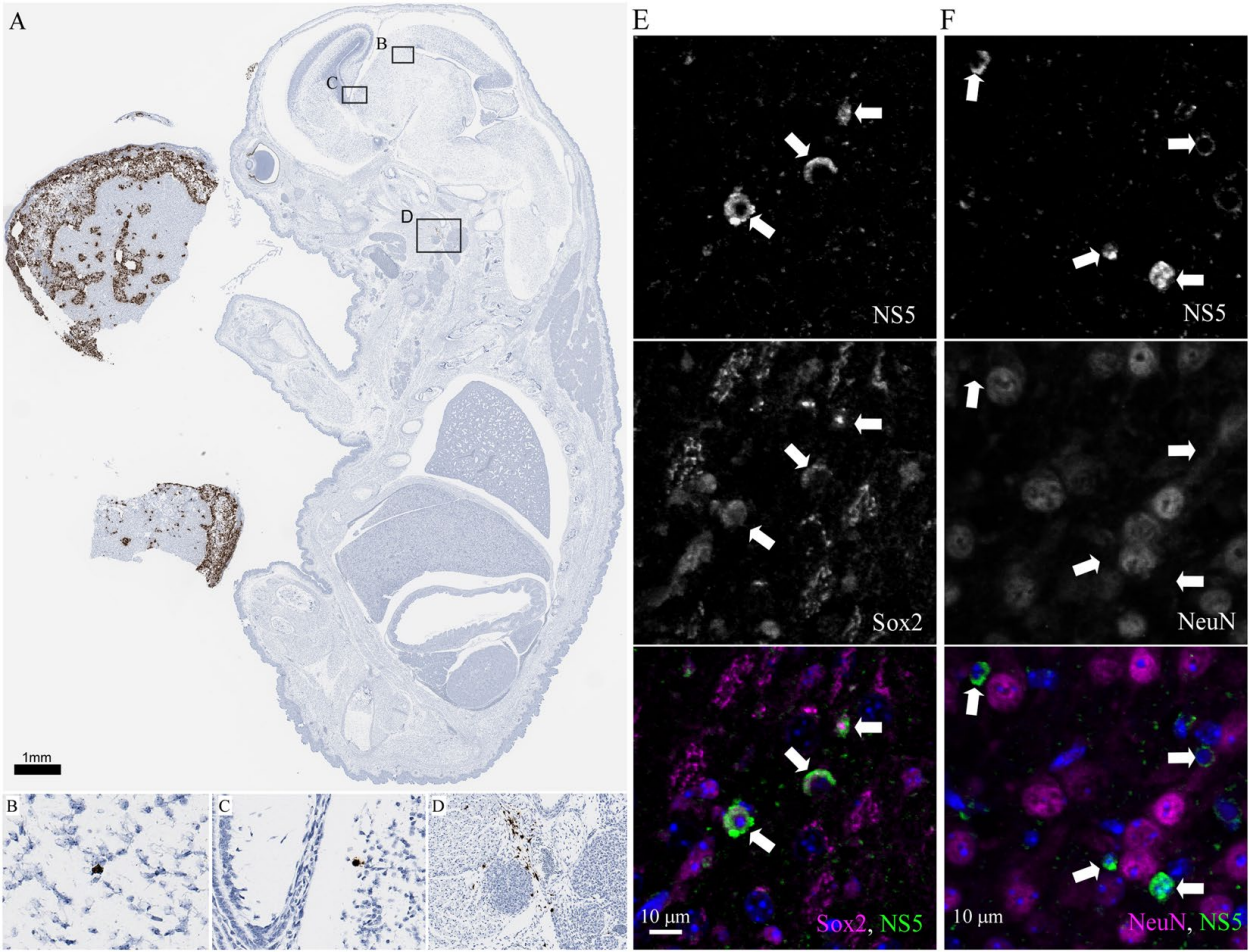
ZIKV infection in pups/fetuses from infected pregnant AIR mice. (**A**) Low magnification image of ZIKV ISH labeled section with nuclear counterstain of a whole-mount fetus (~E18) and associated placenta from an infected, pregnant AIR mouse. Notice the intense labeling in the placenta on the left side of the image. Areas outlined in boxes correspond to high magnification images of ZIKV positive cells in the (**B,C**) pretectum of the brain and the (**D**) pharyngeal/cervical region of the embryo. Dark signal associated with the eye is the developing retinal pigment epithelium and not ZIKV RNA as control animals also contained this signal (not shown). The scale bar shown in (**A**) pertains to (**A–D**). (**E,F**). IHC of medulla tissue from a neonatal pup. Tissue was labeled with antibodies against ZIKV NS5 (green) and co-stained with (**E**) Sox2 (magenta) or (**F**) NeuN (magenta). DAPI stain was used to visualize cellular nuclei. Scale bars are labeled.

Histological and morphological analysis of brains from fetuses/pups demonstrated grossly normal development (Fig. 5A). However, viral RNA (Fig. 5B,C) and antigen (NS5) (Fig. 5E,F) were found in multiple areas of the developing CNS including the neocortical ventricular zone, superior colliculus, pretectum, tegmentum, thalamus, medulla and cervical spinal cord (Fig. 5A–C and data not shown). ZIKV CNS infection was not widespread, but found typically in single or small groups of cells (Fig. 5A–C and E,F). Co-staining demonstrated ZIKV infection was in early neuroprogenitor Sox2^+^ cells (Fig. 5E) and not mature NeuN^+^ neurons (Fig. 5F). Thus, VTx of ZIKV in AIR mice resulted in focal infection of ZIKV in neuroprogenitor cells of the CNS.

Analysis of a whole fetuses/pups showed virus RNA outside the CNS in two of 11 animals, one with infection of the pharyngeal/cervical region (Fig. 5A) and another in a lymph node rostral to the salivary gland (Supplementary Fig. 2A–D). Further analysis using ISH primers that bind replicative RNA intermediates of ZIKV also detected positive signal in the lymph node (Supplementary Fig. 2C), indicating active virus replication and not just phagocytosis of virus particles. IHC for Iba1^+^ macrophages showed strong staining of this region, correlating with the identification as a lymph node (Supplementary Fig. 2D). Although only two of 11 animals had detectable virus outside the CNS, these findings do demonstrate that VTx can result in infection of other fetal tissues besides the CNS.

### ZIKV infection of maternal placental tissue

**Figure 6:**
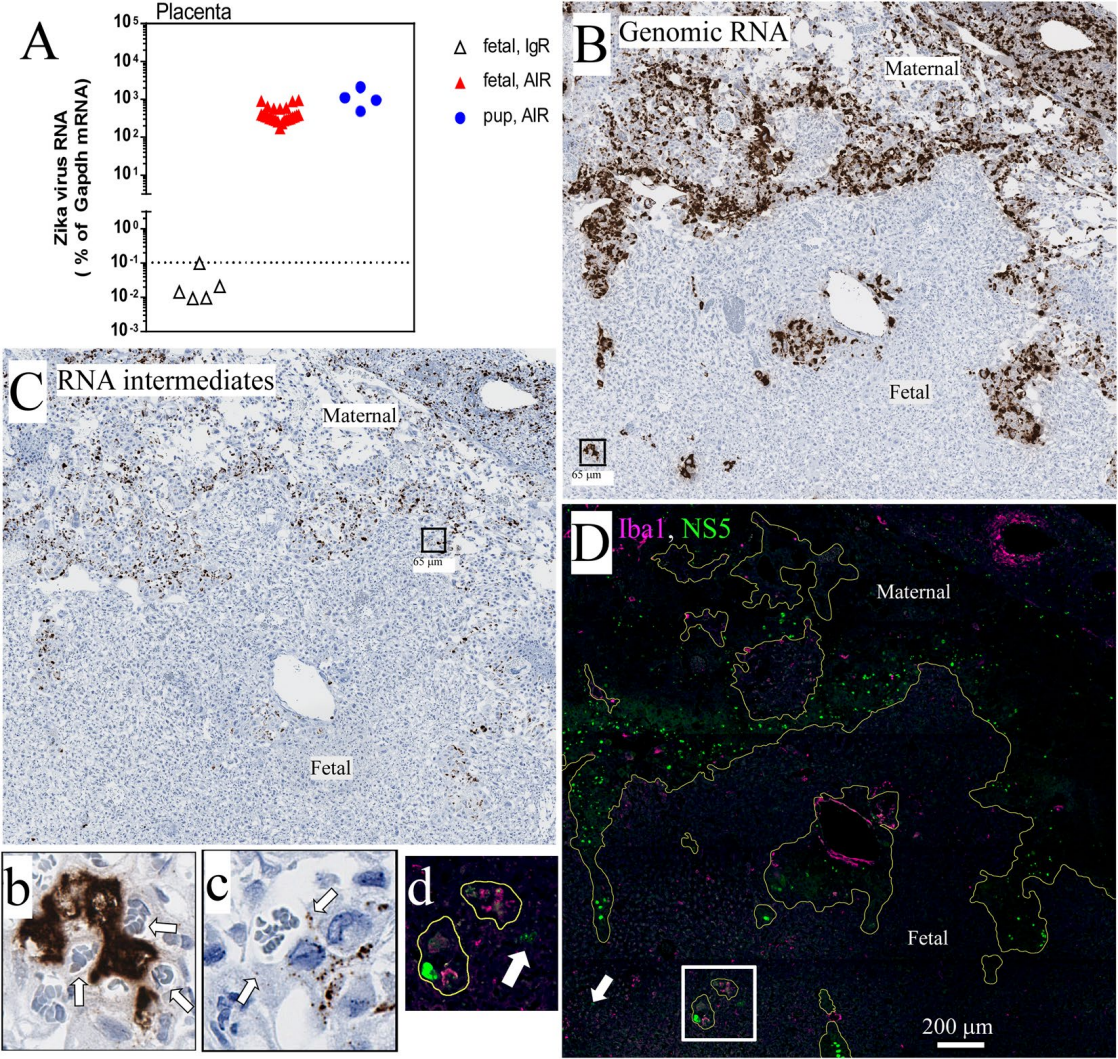
ZIKV infects the placenta in pregnant AIR mice. (**A**) qRT PCR analysis for ZIKV RNA in placental tissues from pregnant, ZIKV infected IgR and AIR mice. Red symbols represent placental tissues from fetal mice, while blue symbols are from newborn pups. (**B–D**) Staining of serial placenta sections from an ~E18 pup from an AIR mouse for (B) ZIKV genomic RNA and (**C**) replicative RNA intermediates by ISH or (**D**) ZIKV NS5 protein (green) and Iba1(magenta) by IHC. (**D**) NS5 labeling is found primarily within the maternal junctional zone (delimited by yellow lines) with a few exceptions (white arrow). Arrows in the insets demonstrate positive ZIKV RNA signal in areas directly opposed to the fetal blood. (b,c) White arrow in (d) represents NS5 staining in fetal placenta. Scale bars are shown as the length of the side of the boxes used to indicate insets (b,c) or are directly shown in the image (**D**).

One of the barriers to fetal infection by ZIKV may be the placenta. Placental tissue from all fetal tissue were analyzed by real-time PCR (Fig. 6A) or IHC and ISH (Fig. 6B–D). Additionally, three placentas were recovered from pups at birth (Fig. 6A) and were used for RNA analysis. All placental tissue from ZIKV-infected AIR mice, regardless if taken pre- or post-birth, were positive for ZIKV RNA, while placental tissue from ZIKV-infected IgR controls were negative (Fig. 6A). ISH and IHC of adjacent sections of placental tissue showed that genomic (Fig. 6B) and replicative intermediates (Fig. 6C) of ZIKV RNA as well as ZIKV NS5 protein (Fig. 6D) were associated with spongiotrophoblast cells of the maternal junctional zone of the placenta (Fig. 6B–D, outlined in 6D). In contrast, virus was largely excluded from fetal placental tissues, with only a few positive signs of ZIKV protein (Fig. 6D and d, white arrows). However, viral RNA was observed in direct apposition to fetal capillary endothelial cells of the labyrinth zone (Fig. 6B–C, inserts). These data suggest active infection and viral replication in areas directly adjacent to the fetal blood supply which is consistent with trans-placental VTx in mice^11^.

### VTx of ZIKV is not associated with breakdown of placental barrier

**Figure 7:**
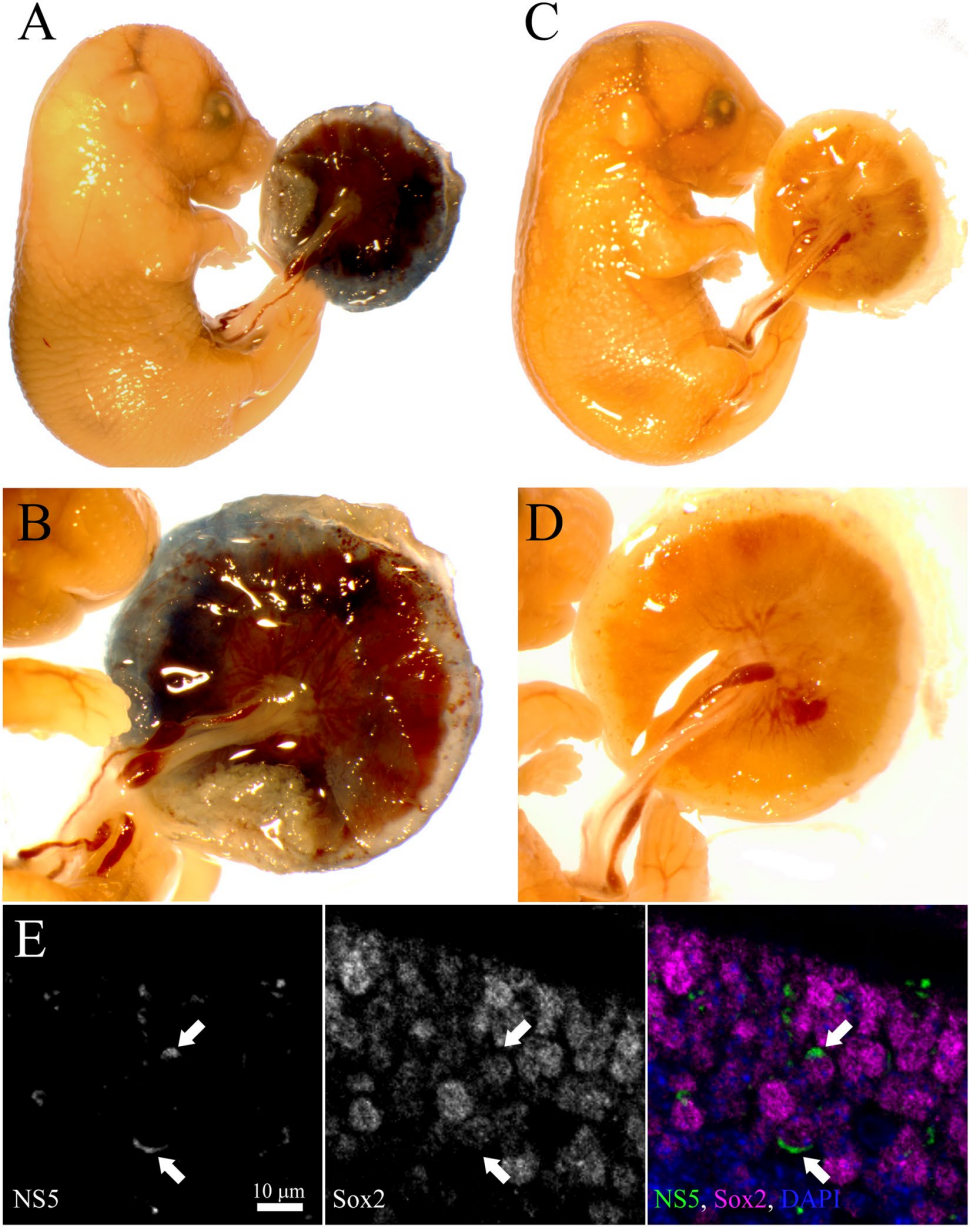
ZIKV infection in AIR mice does not cause extensive placental barrier breakdown. (**A,B**) Gross histological analysis of Evans Blue dye leakage across the placenta into the umbilical cord and fetuses of embryonic day 17–18 ZIKV-infected AIR mice. (**C**,**D**) Non-Evans Blue injected control fetuses from the same embryonic day. (**E**) IHC sections labeled for ZIKV NS5 protein (green), Sox2 (magenta) and DAPI (blue) from the neocortical ventricular zone of the fetus shown in (**A**).

One potential explanation for the VTx of ZIKV in some fetuses and not others within the same dam could be the breakdown of the maternal/fetal barrier in the placenta. To directly examine if this barrier was breached in fetuses with ZIKV infection, we injected three pregnant dams with Evans Blue dye at embryonic day 17–18. Placentas and fetuses from these dams were analyzed for Evans Blue leakage into the umbilical cord and fetus. Interestingly, no Evans Blue dye was detected in the umbilical cord or fetuses by gross histological examination, while the placenta showed clear Evans Blue staining (Fig. 7A,B) compared to untreated controls (Fig. 7C,D). IHC analysis of fetal CNS tissues from some of these fetuses detected ZIKV NS5 staining in Sox2^+^ cells in the neocortical ventricular zone (Fig. 7E), suggesting that virus infection of the fetus occurred in the absence of placental barrier breakdown. Thus, ZIKV is vertically transmitted in AIR mice, but this may be an active process between maternal and fetal cells, rather than a passive leakage due to breakdown of the placental barrier.

## Discussion

ZIKV is an emerging infectious disease that is transmitted to humans by mosquitos as well as human-to-human by VTx and STx^1^. In the current study, we show dam-to-fetus VTx as well as male-to-female STx in AIR mice. VTx was observed in approximately 26% of fetuses/pups, with virus detected in focal areas of Sox2^+^ neuroprogenitor cells in CNS tissue. These results are similar to those observed with other VTx models including ZIKV-infection of *Ifnar1* ^−/−^ mice or SJL mice, but exclude the potential complications of severe weight loss associated with ZIKV-infection of *Ifnar1* ^−/−^ mice^8^ or the high inoculating dose required to overcome the IFN response in SJL mice^15^. Furthermore, the lack of weight loss in pregnant AIR over the time of pregnancy allows for live birth of ZIKV infected pups that can be followed post-birth to examine the long-term consequences of ZIKV-infection. This will allow analysis of how ZIKV infection affects glial cell responses, which develop after birth in the mouse, but in the third trimester in humans^16–18^. Additionally, this study identified two pups that showed replicating virus in the lymph nodes of the developing fetus. These data suggest that CNS tissues are not the only source of virus replication in the developing fetus and that the lymphatic system in the developing fetus may be an additional source of replicating virus.

Infection of the fetus did not appear to directly correlate with virus levels in the dams, as both infected and uninfected fetuses/pups were found in all litters. In contrast, all placentas analyzed were positive for virus, with heavy ISH and IHC labeling on the maternal side. This suggests the maternal/fetal placental barrier is a critical factor in controlling virus spread to the fetus. However, no clear breakdown of the maternal/fetal barrier was observed as determined by Evans Blue dye analysis despite clear VTx of ZIKV to the fetal CNS. The ability of virus to cross to the fetal side of the placenta may be a more random event, depending on active trafficking of virus or infection of cells forming the placental barrier. Further analysis of virus infection of the placentas associated with infected fetuses from varying time points during pregnancy compared to time-matched uninfected fetuses may provide a better understanding of the critical factors that allow virus to invade the fetal side of the placenta and infect the fetus.

The spread of ZIKV to the fetus in AIR mice, which lack adaptive immune cell responses, indicate that neither T cells nor B cells contribute to the spread of ZIKV to the fetus. However, this does not rule out a potential role for these cells in protection against VTx. A recent report demonstrates that the humoral response within amniotic fluid of ZIKV infected pregnant women is higher than during normal pregnancy^19^. These elevated responses, while potentially harmful to the fetus may be critical to preventing VTx of virus^20^. Adoptive transfer studies in the AIR mouse model will allow for analysis of the contribution of specific adaptive immune responses in protection against VTx.

In addition to VTx, AIR mice also transmitted virus sexually. This is only the second model showing direct animal-to-animal transmission of ZIKV by sexual contact^10^. The ability to clearly demonstrate STx in AIR mice provides a model for investigating mechanisms of transmission as well as testing of candidate therapies to inhibit this process. One interesting finding was the ability of some of the *Ifnar1* ^−/−^females to control virus infection following STx. The ability of these mice to control virus may be due to dose of virus transmitted from the males. In another study, *Ifnar1* ^−/−^mice inoculated intravaginally with a low dose of ZIKV only lost a modest amount of weight, while those infected with a high dose developed severe wasting disease^21^. Similarly, the mice that recovered in our studies may have received a lower dose of virus. Surprisingly, *Ifnar1* ^−/−^ mice that recovered from weight loss had similar NAb response to *Ifnar1* ^−/−^ mice that did not recover, suggesting that the development of NAb response was not the key mediator of recovery. Thus, other components of the innate or adaptive immune response may provide the critical protection to control virus spread even in the absence of the type I IFN response.

In the previous study showing STx of ZIKV in IFN α/β and -γ receptor deficient mice the amount of infectious virus decreased in the testes of mice later in infection despite the persistence of viral RNA^10^. These findings are similar to the current findings as infected male AIR mice were able to efficiently transmit virus sexually between 8–12 dpi, but to a lesser extent between 12–16 dpi. Thus, there appears to be a time frame following ZIKV infection in males, where virus can be transmitted. The lack of virus transmission in the later mating of these AIR mice could be due to several reason, including decreased mating frequency in male mice due to weight loss or preference on the part of the female to not mate with an infected male^22^. Additionally, ZIKV-infects spermatozoa and mature sperm^7, 23^, and these cells decrease in number in mice at later stages of ZIKV infection^24^. Thus, the time frame for transmission may be after infection in the testes, but prior to wide spread death of spermatogonia or adult sperm. In contrast, ZIKV RNA can persist in semen for many months in humans^7^. It is possible the innate and adaptive immune response may sufficiently control infection to prevent widespread cell death in human testes, but not sufficient prevent these cells from becoming infection. Further investigation of this AIR model will define if there is a critical time frame of sexual transmission in these mice, how timing correlates with infection and apoptosis of spermatogonia in the testes, and the role of immune cells in ZIKV STx.

## Methods

### Treatment and infection of mice with ZIKV

All animal work was conducted in compliance with the guidelines of and under a protocol approved by the corresponding Institutional Animal Care and Use Committee (IACUC, Protocol #: 2016-015). All applicable international, national, and/or institutional guidelines for the care and use of animals were followed.” *Rag1* ^−/−^ mice which are deficient in B and T lymphocytes and *Ifnar1* ^−/−^ mice which are deficient in the type I interferon receptor, (Jackson Laboratories) were maintained on a C57BL/6 background in a breeding colony at RML. The 2015 Zika Paraiba strain is a human microcephaly isolate that has been previously described^9^ and was kindly provided by Steve Whitehead (NIAID). Experimental mice were inoculated with 10^4^ PFU of ZIKV, diluted in PBS, i.p. in a volume of 200 μl/mouse.

AIR mice were either male or female *Rag1* ^−/−^ mice (as required for the transmission route) treated i.p. with 1 mg of anti-IFNAR1 clone MAR1-5A3. Male mice used for STx studies received injections on −1, 1, 3 and 7 dpi. Pregnant female mice used for VTx studies received injections on −1, 1, 3, 7 and 11 dpi. IgR control mice were either male or female *Rag1* ^−/−^ mice (as required for the transmission route) treated with an equivalent amount of normal mouse IgG on the same schedules described above.

For STx experiments, 8–10 week old ZIKV infected male AIR mice were bred to age-matched naïve *Ifnar1* ^−/−^ female mice for four days at either 8–12 or 12–16 dpi. Infected AIR female mice were cohoused with naïve *Ifnar1* ^−/−^ female mice at the same dpi to control for non-sexual virus transmission. Infectious AIR mice were cohoused with two *Ifnar1* ^−/−^ female mice per cage for the four-day transmission period and then recipient mice were housed individually and followed for weight loss or signs of disease.

For VTx experiments, 8–10 week old *Rag1* ^−/−^ female mice were time-mated with age-matched males of the same genotype for 3 days. At 6 dpm, pregnancy was estimated via ultra-sound and/or abdominal palpation and anti-IFNAR1 or control IgG was administered as designated in Fig. 4A to generate female AIR mice. Pregnant AIR mice were infected at 7 dpm i.p. with 10^4^ PFU ZIKV and individually housed upon infection such that pups taken post-birth could be identified and compared to littermates.

Infected mice were observed daily for signs of neurological disease including hunched posture, seizures, reluctance or inability to move normally or paralysis. Mice were also weighed every 2–3 days to measure weight loss. Animals with signs of neurological disease or greater than 20% loss of original starting weight were scored as clinical and euthanized immediately.

### Immunohistochemistry and *in situ* hybridization

For VTx studies, fetuses and pups were fixed in 10% neutral buffer formalin for 2–3 days, at which time the whole fetus/pups was cut in half sagittally along the midline and returned to fix for an additional week. Whole fetuses/pups were serially sectioned (5μm) and mounted on slides. For IHC, sections were processed and imaged as previously described^25^. Antibodies against ZIKA NS5 (1:3000, Aves Labs), and NeuN (1:2500, abcam) or Sox2 (1:750, abcam) or Iba1 (1:250, Dako) or active Caspase 3 (1:250, Promega) were used at the indicated concentrations. Secondary antibodies (goat anti-chicken AF488, 1:500 and donkey anti-rabbit AF594, 1:500) were used to label specific primaries. For transmitted light staining, pan-flavivirus-4G2 (1:100, EMD Millipore) primary and goat anti-rabbit HRP (1:500) secondary were used.

*In situ* hybridization was performed using sense and anti-sense riboprobes that target multiple ZIKV genes as previously described^26^. All riboprobes were designed and tested by Advanced Cell Diagnositics (Newark, Ca). ZIKA genomic RNA was detected using an anti-sense probe (complementary to positive sense strand) and replicative RNA intermediates were detected using a sense probe. Resulting staining was imaged using at 40X using an Aperio XT (bright field) slide scanner.

### Viremia and NAb detection

Detection of viremia and NAb was done as previously described^12^. Briefly, for detection of viremia and virus in the testes, serial dilutions of plasma or clarified tissue homogenate were plated directly to Vero cells as described below. Testes were homogenized in 500 ul of PBS using a Precellys tissue homogenizer and clarified by centrifugation. For quantification of NAb, serially diluted plasma was mixed with 10^2^ PFU of ZIKV and incubated for neutralization for 1hr and then added to on confluent Vero cells in a 24-well plate for 1 hr. Cells were then overlaid with 1.5% carboxymethyl cellulose in MEM, cultured for 5 days and then fixed with 10% formaldehyde. After fixation, plates were rinsed and stained with 0.35% crystal violet to reveal plaques. Viremia was calculated by dividing the number of plaques per sample by the plasma dilution factor multiplied by the volume of each well. Neutralizing titer was determined by the dilution that inhibited at least 50% of plaque formation when compared to cells infected with the 10^2^ ZIKA.

### Detection of ZIKV by vaginal swab

*Ifnar1* ^−/−^ female mice received vaginally swabs immediately prior to mating with infected male AIR mice, and again 5, 10 and 14 dpm unless they developed clinical disease, in which case a swab was taken immediately before euthanasia. Vaginal swabs were obtained using a sterile polyester tipped applicator (Puritan Medical Products) inserted into the vagina of the restrained mouse. Once inserted, the swab was gently rolled between the index finger and thumb of the investigator a total of 12 times to collect a sample from the vaginal wall. The tip of the swab was immediately transferred to a microcentrifuge tube containing 200 μl of Viral RNA buffer (Zymo) where it was stored at −20 °C at least overnight. Tips were then inverted in the microcentrifuge tube and spun at 5000 g for 5 min to remove buffer from the tip. Sample were then prepared for real-time PCR as described below.

### Real-time PCR

qRT PCR analysis of mRNA expression from brain, spleen and testes was completed as previously described^27^. The primers used include: Gapdh.2-152F (TGCACCACCAACTGCTTAGC), Gapdh.2-342R (TGGATGCAGGGATGATGTTC), ZIKAFP8008F (AAGCTGAGATGGTTGGTGGA) and ZIKAFP8121R (TTGAACTTTGCGGATGGTGG). Primers were subjected to BLAST analysis (NCBI) to ensure detection of only the specified gene and were tested on positive controls to ensure amplification of a single product. Data for each sample were calculated as the percent difference in threshold cycle (*C*_*T*_) value (Δ*C*_*T*_ = *C*_*T*_ for GAPDH gene – *C*_*T*_ for specified gene). Gene expression was plotted as the percentage of gene expression relative to that of the GAPDH gene.

### Evans Blue injection of infected pregnant AIR mice

To examine vascular leak across the placenta during ZIKV infection, Evans Blue dye was administered intravenously to pregnant AIR mice at embryonic day 17–18 as previously described^25^. Following injection, whole fetuses were dissected and fixed with 10% neutral buffered formalin. Whole pups were imaged with an Olympus SZX16 dissection scope coupled to an Olympus DP25 camera. Following whole pup imaging, samples were cut in half sagittally and sectioned for IHC and confocal microscopy as described above.

### Statistical analysis

All statistical analyses were performed using Prism software Version 7.01 (GraphPad). Statistical tests used included One-way or Two-way ANOVA with Tukey post-hoc multiple comparisons. Significance threshold was set at all P < 0.05. * in figures indicates P < 0.05, ** indicates P < 0.01.

### Availability of Materials and Data

All data generated or analyzed during this study are included in this published article or its supplementary information files.

## Electronic supplementary material

Supplementary Information 
