# Supplementary material for: Sexual and Vertical Transmission of Zika Virus in anti-interferon receptor-treated Rag1-deficient mice
Source: Sci Rep. 2017 Aug 3;7:7176. doi: 10.1038/s41598-017-07099-7 (PMC5543051; doi:10.1038/s41598-017-07099-7)
Supplement: Supplementary file 1 — Supplementary Information [file 41598_2017_7099_MOESM1_ESM.doc]

**Supplementary Information**

**Sexual and Vertical Transmission of Zika Virus in anti-interferon receptor-treated Rag1-deficient mice**

Clayton W. Winkler, Tyson A. Woods, Rebecca Rosenke, Dana P. Scott, Sonja M. Best and Karin E. Peterson

**Supplementary Figures**

**Supplementary Figure S1**. Weights and NAb titers from *Ifnar1^-/-^* mice that were mated with ZIKV infected AIR males at 8-12 dpi and became pregnant and mice mated with AIR males at 12-16 dpi. A) Color-coded individual plots of percent (%) starting body weight plot for 3 *Ifnar1^-/-^* female mice that were impregnated when bred to infectious AIR males between 8-12 dpi (A). (B) Inhibitory dilution of ZIKV NAb from the three pregnant mice shown in (A). (C) Individual plots of percent (%) starting body weight of *Ifnar1^-/-^*  female mice bred with infected male AIR mice (black tracings), bred with infected male AIR mice but gain no weight (green tracing), impregnated by infected AIR male mice (red tracing) or co-housed with ZIKV-infected female AIR mice (blue tracings) between 12-16dpi. (D) Inhibitory dilution of ZIKV NAb from the *Ifnar1^-/-^* mice shown in (C).


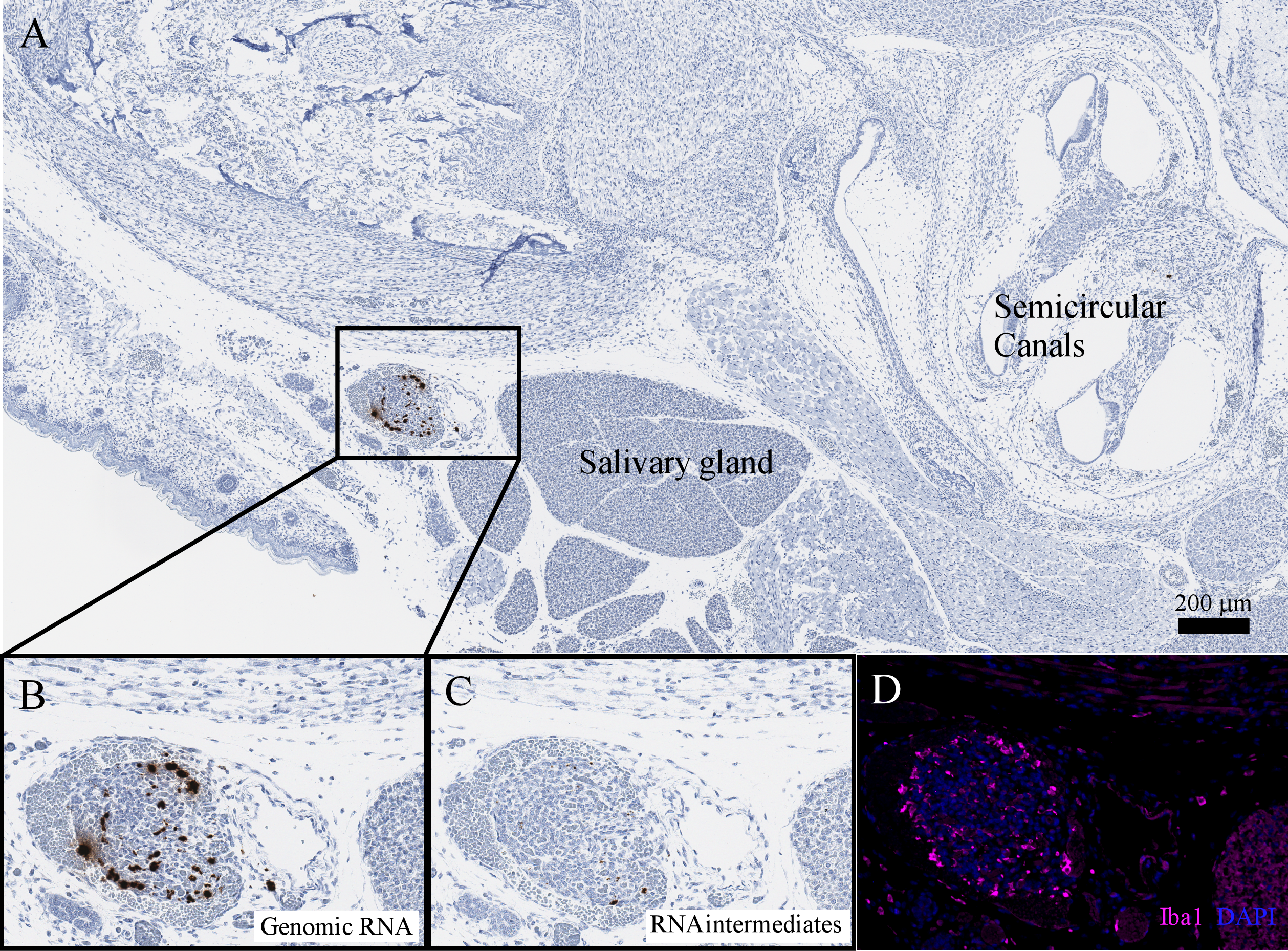


**Supplementary Figure S2**. Vertically transmitted ZIKV infects and replicates in cells of the cervical/submandibular lymph node in pups born to ZIKV-infected AIR mice. The pup shown is from the same cohort shown in Fig. 5. (A) Low magnification image of ISH staining for ZIKV genomic RNA with a nuclear counterstain in the cervical/submandibular region from a new born mouse whole skull prep. Positive viral RNA signal is evident in the cervical lymph node (black box). The semicircular canals of the ear and the salivary gland are labeled for reference. Scale bar length is as indicated (B-D) Higher magnification images of the same cervical lymph node shown in (A) with ISH labeling from (B) genomic RNA, (C) replicative RNA intermediates and immunohistochemical labeling for (D) Iba1 (magenta) to label monocyte/macrophages commonly found in lymph node.
